# Editorial: Molecular surveillance of animal and zoonotic pathogens

**DOI:** 10.3389/fvets.2024.1466949

**Published:** 2024-07-30

**Authors:** Víctor Martínez, Indira Kudva, Rodrigo J. Nova, Nilton Lincopan, Nicolás Galarce

**Affiliations:** ^1^Departamento de Fomento de la Producción Animal, Facultad de Ciencias Veterinarias y Pecuarias, Universidad de Chile, Santiago, Chile; ^2^Food Safety and Enteric Pathogens Research Unit, National Animal Disease Center, Agricultural Research Service, United States Department of Agriculture, Ames, IA, United States; ^3^School of Biodiversity, One Health and Veterinary Medicine, College of Medical, Veterinary and Life Sciences, University of Glasgow, Glasgow, United Kingdom; ^4^Departamento de Microbiología, Instituto de Ciências Biomédicas, Universidade de São Paulo, São Paulo, Brazil; ^5^Departamento de Medicina Preventiva Animal, Facultad de Ciencias Veterinarias y Pecuarias, Universidad de Chile, Santiago, Chile

**Keywords:** zoonoses, surveillance, pathogen, epidemiology, animals

## Introduction

Bacteria, fungi, viruses, and parasites can threaten the health and welfare of all terrestrial and aquatic animal species, including all domestic and wildlife animals. This may lead to high rates of morbidity and mortality, increased socioeconomic costs, food insecurity, and biodiversity loss (1, 2). Furthermore, many of these pathogens are zoonotic or have zoonotic potential, thus posing a threat to global public health (3).

Due to globalization, there is an increased risk of emergence and spread of pathogens, resulting in a higher likelihood of outbreaks caused by new pathogens, and/or pathogens previously absent in an area/region (4). Therefore, understanding the molecular epidemiology of these microorganisms, including their geographical distribution, virulence, resistance determinants, genomic patterns, and the effects of climate change and anthropogenic activities is essential.

Genomic changes of infectious agents relate to microbial evolution. These changes often impact pathogenicity, virulence, dissemination capacity, and resistance to intervention and prevention methods such as use of antimicrobials and vaccines. Additionally, they could pose a challenge to current diagnostic tools (5). Under this scenario, there is a need for reviewing current prevention and control measures, to establish an integrated approach, considering transdisciplinary collaborations, following the One Health concept.

This Research Topic presents five new articles that shed light on these issues.

## Wild animals as reservoirs of zoonotic pathogens

Wang et al. presents the prevalence and genetic composition of *Blastocystis* in wild rodents and shrews in China, revealing that these animals can harbor zoonotic subtypes of *Blastocystis*. Similarly, Ardila et al. investigates the presence of *Borrelia, Anaplasmataceae*, and Piroplasmida in cricetid rodents of Central and Southern Chile. Although no pathogens were detected; the authors suggested they may be present at a very low prevalence, emphasizing the need for further research to understand the factors influencing the presence of these agents and their vectors. Liu et al. provides an insight into the presence and possible dissemination of the Jingmen tick virus from ticks obtained from wild boars in China, suggesting complex transmission routes and providing valuable information on its distribution and evolution in that country.

## Antimicrobial resistance

Domán et al. investigates the genomic differences between avian and human *Candida albicans* isolates with a particular focus on antifungal resistance genes. Their results suggest that the use of environmental fungicide might exert selective pressure on *C. albicans* infecting animals, thus contributing to the spread of potentially resistant strains. In a similar context, Bazalar-Gonzales et al. assesses the presence of extended-spectrum beta-lactamase-producing *Escherichia coli* strains in captive and semi-captive non-human primates from the Peruvian Amazon and analyze their genomes, reporting strains closely related to high-risk pandemic lineages found in humans and domestic animals, highlighting the negative impact of anthropogenic activities on Amazonian wildlife.

Overall, this Research Topic represents an important body of knowledge that compiles several aspects of pathogen emergence considering a range of animals and pathogens. These findings may help to understand the emergence of pathogens at the human-animal-environment interface and provide relevant information for optimizing diagnostic methods in these changing times.

## Publishers' note

All claims expressed in this article are solely those of the authors and do not necessarily represent those of their affiliated organizations, or those of the publisher, the editors and the reviewers. Any product that may be evaluated in this article, or claim that may be made by its manufacturer, is not guaranteed or endorsed by the publisher.

## Author contributions

VM: Writing – original draft, Writing – review & editing. IK: Writing – original draft, Writing – review & editing. RN: Writing – original draft, Writing – review & editing. NL: Writing – original draft, Writing – review & editing. NG: Writing – original draft, Writing – review & editing.
